# Dopamine DRD2 and DRD3 Polymorphisms Involvement in Nicotine Dependence in Patients with Treatment-Resistant Mental Disorders

**DOI:** 10.3390/jpm12040565

**Published:** 2022-04-02

**Authors:** Antonio del Casale, Marco Paolini, Giovanna Gentile, Marina Borro, Clarissa Zocchi, Federica Fiaschè, Alessio Padovano, Teodolinda Zoppi, Martina Nicole Modesti, Ottavia De Luca, Leda Marina Pomes, Roberto Brugnoli, Stefano Ferracuti, Paolo Girardi, Maurizio Pompili, Maurizio Simmaco

**Affiliations:** 1 Department of Dynamic and Clinical Psychology, and Health Studies, Faculty of Medicine and Psychology, Sapienza University, Via degli Apuli 2, 00185 Rome, Italy; paolo.girardi@uniroma1.it; 2 Unit of Psychiatry, ‘Sant’Andrea’ University Hospital, Via di Grottarossa, 00189 Rome, Italy; marcopao88@gmail.com (M.P.); clarissa93.zocchi@gmail.com (C.Z.); fiaschefederica@libero.it (F.F.); alessio.padovano@uniroma1.it (A.P.); teodolindazoppi@gmail.com (T.Z.); martinanicolemodesti@gmail.com (M.N.M.); roberto.brugnoli@uniroma1.it (R.B.); maurizio.pompili@uniroma1.it (M.P.); 3 Department of Neuroscience, Mental Health and Sensory Organs (NESMOS), Faculty of Medicine and Psychology, Sapienza University, Via di Grottarossa, 00189 Rome, Italy; giovanna.gentile@uniroma1.it (G.G.); marina.borro@uniroma1.it (M.B.); ottavia_deluca@yahoo.it (O.D.L.); ledama@hotmail.it (L.M.P.); maurizio.simmaco@uniroma1.it (M.S.); 4 Unit of Laboratory and Advanced Molecular Diagnostics, ‘Sant’Andrea’ University Hospital, Via di Grottarossa, 00189 Rome, Italy; 5 Department of Human Neuroscience, Sapienza University, Viale dell’Università 30, 00185, Rome, Italy; stefano.ferracuti@uniroma1.it; 6 Unit of Risk Management, ‘Sant’Andrea’University Hospital, Via di Grottarossa, 00189 Rome, Italy

**Keywords:** nicotine dependence, mood disorders, schizophrenia-spectrum disorders, treatment-resistant mental disorders, *DRD2*, *DRD3*

## Abstract

Patients affected by mental disorders smoke more than the general population. The reasons behind this habit are genetic, environmental, etc. This study aims to investigate the correlations between some polymorphisms and the smoking habits and nicotine dependence in patients with psychiatric disorders. We recruited 88 patients with treatment-resistant mental disorders, including 35 with major depressive disorder, 43 with bipolar spectrum disorder, and 10 with schizophrenia spectrum disorder. We carried out a clinical and psychometric assessment on current smoking habits, years of smoking, number of daily cigarettes, and level of nicotine addiction. The patients performed a peripheral blood sample for DNA analyses of different polymorphisms. We searched for correlations between the measures of nicotine addiction and analysed genotypes. The expression of the T allele of the *DRD2 rs1800497* and *DRD3 rs6280* polymorphisms significantly correlated with a lower level of nicotine dependence and lower use of cigarettes. We did not find significant correlations between nicotine dependence and *OPRM1 rs1799971, COMT rs4680* and *rs4633* polymorphisms, *CYP2A6 rs1801272* and *rs28399433*, or *5-HTTLPR* genotype. Concluding, *DRD2 rs1800497* and *DRD3 rs6280* polymorphisms are involved in nicotine dependence and cigarette smoking habits in patients with treatment-resistant mental disorders

## 1. Introduction

Cigarette smoking is one of the main causes of morbidity and mortality worldwide. Tobacco is considered an important cause of non-heritable disease in the world, producing even more deaths than overweight [1]. From 2000 to 2015, there has been a progressive decrease in tobacco use in both sexes and all age groups. However, there are approximately one billion tobacco smokers in the world [2]. The literature has shown that patients with different mental disorders smoke cigarettes more frequently than the general population. For example, an Australian survey found that smokers percentage among psychiatric patients is double than that of healthy individuals [3,4]. Although psychiatric patients smoke more, have a higher level of nicotine dependence, and a lower quit rate than the general population [5,6], those who are properly treated for their mental disease have a higher smoking quitting rate [7]. The reasons for this widespread habit to smoke cigarettes among psychiatric patients can be many. Smokers and patients with psychiatric diseases share a common genetic predisposition. Environmental factors and various stressors seem to increase the risks of both starting smoking and developing mental illness.

In some cases, patients try to “self-medicate” with nicotine, although smoking could increase the levels of anxiety and depression [3]. By examining individual mental illnesses and their relationship with cigarette smoking, we can appreciate differences between diagnoses. For example, the smoking rate among depressed patients is almost double than that of the general population [8]. Although there are several reasons why people suffering from depression tend to smoke more and develop nicotine addiction, many patients with a depressive disorder are highly motivated to quit, and they can achieve a prolonged period of tobacco abstinence. However, withdrawal syndrome can cause depressive symptoms during the first two weeks of quitting. Therefore, there could be a two-way relationship in which cigarette smoking addiction and depressed mood can influence each other [9]. As far as other mental diseases are concerned, the prevalence of nicotine addiction in patients with bipolar disorder is higher than that of patients with depression and lower than that of patients with schizophrenia [10], who are still three times more likely to start smoking than the general population [11].

Patients with treatment-resistant depression (TRD) show a high smoking rate. Active tobacco cessation should be achieved in TRD to improve depressive and impulsive symptoms, and reduce the risk of suicide, especially in women [12]. Other serious clinical issues concern patients with treatment-resistant schizophrenia. In these patients, nicotine dependence was associated with more severe impairments in cognitive functioning, negative symptoms, and social adjustment, reflecting a possible worsening of neuronal dopamine dysfunction induced by nicotine [13].

Nicotine acts on cholinergic nicotinic receptors (α_4_β_2_ is one of the forms mainly involved in addiction mechanisms) in different brain areas. This bond causes the release of various neurotransmitters, including dopamine. Dopaminergic neurons involved in the development of nicotine addiction (responsible for pleasure and reward) are those located in the ventral tegmental area (VTA) and in the shell of the nucleus accumbens [14]. A recent meta-analysis conducted on adolescents aged 13 to 19 years old reported how environmental and genetic factors can influence young people to start smoking in different periods of adolescence. Environment acts in the first years mostly, while genetic factors play a role in the last part of this very delicate period [15]. The genes involved in the development of nicotine addiction have been extensively studied in the literature, but only three of these are currently universally recognized as such. These are clusters of genes that code for nicotinic cholinergic receptor subunits (CHRNA5/A3/B4 and CHRNB3/A6) and the cytochromes responsible for nicotine metabolism (CYP2A6/2A7) [16]. A recent GWAS study analysed many genes associated with tobacco and alcohol use in a sample of 1.2 million people. In addition to confirming the data already present about CYP2A6 and nicotinic acetylcholine receptors, it obtained statistically significant results for the DRD2 (associated with both alcohol and cigarettes use) and GRK4 (a kinase involved in the functioning of several receptors including DRD3) genes [17]. Since dopamine plays a key role in the reward pathway, and consequently in smoking habit and the development of nicotine addiction, several genes coding for its receptors and metabolites have been extensively studied. These include the DRD2 [16,18,19], DRD1, DRD3 [20,21] and DRD4 [22,23], COMT [24], and DOPA decarboxylase [25].

Other studies focused on the involvement of the *SLC6A4/5-HTTLPR* (serotonin transporter) gene in the pathophysiology of nicotine dependence. Although some studies have associated this gene with smoking habit, two meta-analyses did not confirm this finding [26,27].

Finally, there is evidence that *OPRM1rs1799971* is associated with an increased risk of developing an addiction to abuse substances. In particular, correlations between the G allele and cigarette smoking have been demonstrated [28,29]. Currently, no studies aimed at examining the relationship between *DRD2, DRD3, COMT*, and *OPRM1* polymorphisms, the *5-HTTLPR* genotype, and smoking habit and nicotine addiction in patients with treatment-resistant mood disorders and schizophrenia-spectrum disorders. We hypothesized that the *ANKK1-DRD2 rs1800497*, *DRD3 rs6280*, *COMT rs4633*, and *OPRM1 rs1799971* polymorphisms, together with the *5-HTTLPR* genotype, could be involved in the smoking habits and nicotine dependence in patients with treatment-resistant psychiatric disorders. The main objectives of this study are to investigate the correlations between these polymorphisms and nicotine addiction in subjects with drug-resistant mental disorders. Research in this area could open the way for new treatment strategies and personalized care.

## 2. Materials and Methods

The study was conducted during 2018–2021 at the Centre of Personalized Medicine and Service of Personalized Mental Health and Pharmacogenomics, Unit of Psychiatry, Sant’Andrea University Hospital, Sapienza University, Rome. We obtained written consent from all participants after fully informing them about the type and aims of the study. This study was carried out following the Principles of Human Rights adopted by the World Medical Association (WMA) at the 18th WMA General Assembly, Helsinki, Finland, June 1964, and subsequently amended by the 64th WMA General Assembly, Fortaleza, Brazil, October 2013.

The inclusion criterion was having received a diagnosis of TRD or treatment-resistant schizophrenia. We defined TRD as a depressive episode with a lack of treatment response after administering two or more trials with antidepressant drugs belonging to different pharmacological classes and administered at appropriate dosages and periods, according to the most accepted definition in the scientific and clinical communities [30]. We defined treatment-resistant schizophrenia as a clinical picture that did not respond to at least two subsequent antipsychotic trials administered at sufficient dosage, with an adequate treatment time, during which the patient properly adhered to therapy [31]. 

Exclusion criteria included minor (≤18) or advanced age (≥75 years), concurrent substance use disorders (except nicotine dependence), neurological illnesses, and severe organic illnesses. All patients were on medications for at least 12 months, with at least three subsequent different standard antidepressant and/or antipsychotic treatments to which they were poorly responsive or unresponsive. We have carried out a clinical and psychometric evaluation to investigate different variables: currently being a smoker, past smoking habits, age of smoking onset, and the number of daily cigarettes. To assess the level of nicotine addiction, we administered a validated and widely used scale, i.e., the Fagerstrom Test for Nicotine Dependence (FAG) [32], which gives rise to a dimensional score (ranging from 0 to 10) relating to nicotine addiction. The Advanced Molecular Diagnostics (DiMA) Unit collected patients’blood samples at. DNA was extracted from peripheral blood using an automated nucleic acid extraction system (Qiasymphony, Qiagen, Hilden, Germany). DNA polymorphisms were analyzed by Next Generation Sequencing using the IonS5 platform (ThermoFisher Scientific, Waltham, MA, USA) and the Ion AmpliSeq™ Library Kit 2.0 reaction chemistry, following the supplier’s instructions (ThermoFisher Scientific, Waltham, MA, USA).

### Statistical Analyses

We used Pearson’s chi-square test to analyse between-group differences in gender composition. We analyzed the dimensional variables (age, diagnosis, sex, number of cigarettes smoked in a day, current or former smoker status and level of smoking addiction), assessing the differences among the diagnostic groups with the T-test for independent samples. We assessed the equality of variances with Levene’s test. We considered the *DRD2 rs1800497*, *DRD3 rs6280*, *5-HTTLPR*, *CYP2A6 rs1801272* and *rs28399433*, and *OPRM1 rs1799971* polymorphisms. We performed a T-test for independent samples with the corresponding genotypes as between-group dependent variables, and the age of onset of smoking, the daily number of smoked cigarettes, and level of nicotine addiction were assessed with the FAG scale as independent variables. We defined slower nicotine metabolizers the individuals expressing the A allele of the CYP2A6 rs1801272 or the G allele of the rs28399433. The cut-off for statistical significance was set at *p* < 0.05. All *p* values were two-tailed. We used the IBM SPSS Statistics 25.0 (Armonk, NY, USA, IBM Corporation, 2017) for all analyses.

## 3. Results

We recruited 88 Caucasic patients with treatment-resistant mental disorders, including 30 males and 58 females. The mean age of the subjects was 48.32 years (SD = 15.47). Study participants were affected by treatment-resistant major depressive disorder (MDD) (35 patients; 23 women, 12 men; mean age 46.2; SD = 18.24), bipolar spectrum disorder (BD) (43 patients; 29 women, 14 men; mean age 51.42; SD = 13.69), and schizophrenia spectrum disorder (SCZ) (10 patients; 6 women, 4 men; mean age 42.4; SD = 8.9). The study groups did not significantly differ in gender composition. The diagnostic groups did not differ in terms of age, history of cigarette smoking, or age at first cigarette use. The SCZ group, compared to the BD, consumed a significantly higher number of daily cigarettes (t = 2.472; *p* = 0.017), showed a higher FAG score (t = 2.507; *p* = 0.015), and were more often current smokers (*χ*^2^ = 7.42; *p* = 0.006). Levene’s test for the equality of variance showed a normal data distribution.

In the sample, 59.1% of patients were on antidepressant therapy, 47.7% on antipsychotics, 34.1% on antiepileptics, 58% on benzodiazepines, and 25% on lithium. There were no significant differences between patients on antipsychotic and non-antipsychotic therapy in terms of years of smoking (t = 1.563; *p* = 0.122), daily cigarette consumption (t = 0.301; *p* = 0.764), and FAG score (t = 0.085; *p* = 0.933). There were no significant differences in patients taking other medications, except for the lithium therapy group, which showed a trend of reduced FAG score, compared to the patients not taking lithium (t = 1.749; *p* = 0.084). We summarized the sociodemographic and clinical characteristics of our sample in Table 1. 

For each polymorphism included, we compared the expression vs. absence of each allele concerning the variables under study, i.e., years of nicotine addiction, number of cigarettes per day, and level of addiction according to the FAG scale. 

*DRD2 rs1800497*. Subjects with expression of the T allele compared to individuals without the T allele showed significantly lower years of nicotine dependence (t = 1.99; *p* = 0.05), daily number of cigarettes (t = 2.431; *p* = 0.017), and lower FAG score (t = 2.1; *p* = 0.039).

*OPRM1 rs1799971*. We did not find between-group differences in terms of age of smoke onset, the daily number of cigarettes, and FAG score in the whole sample. 

*COMT rs4680* and *rs4633*. We did not find between-group differences in age of smoke onset, daily number of cigarettes, and FAG score.

*DRD3 rs6280*. Subjects with expression of the T allele, compared to individuals not expressing the T allele, showed a tendency of lower years of nicotine dependence (t = 1.763; *p* = 0.082), significantly lower daily number of cigarettes (t = 2.111; *p* = 0.038), and lower FAG score (t = 2.335; *p* = 0.022). 

*5-HTTLPR*. We did not find between-group differences in terms of age of smoke onset, daily number of cigarettes, and FAG score.

*CYP2A6 activity reduction*. We did not find between-group differences in terms of age of smoke onset, daily number of cigarettes, and FAG score.

We summarized the main findings in Table 2 (significant results) and Supplementary Table S1 (all results).

## 4. Discussion

This study sought to explore the role of the DRD2 rs1800497 and DRD3 rs6280 polymorphisms in nicotine addiction among patients with treatment-resistant mood disorders and schizophrenia spectrum disorders. In the study sample, we found no effect of drug treatments and CYP2A6 polymorphisms on years of nicotine addiction, the number of cigarettes per day, and the level of addiction. Patients with treatment-resistant mental disorders are often on chronic antipsychotic treatments. A chronic treatment with dopamine D2 receptor antagonists has been proposed to lead to postsynaptic increases in D2 receptor expression [33]. Also considering this phenomenon, the lack of differences in the measures of nicotine dependence between patients on antipsychotics acting on dopaminergic receptors, and patients not taking antipsychotic therapy, supports the findings, and suggests that antipsychotic therapy does not influence nicotine addiction. Although further studies are needed on this topic, we found that a different and protective role could instead be played by lithium.

*DRD2 rs1800497.* In our sample, the T allele was protective regarding nicotine dependence. This is in line with the existing evidence on the involvement of DRD2 rs1800497 in nicotine dependence in outpatients affected by cancer [34], in outpatients visited for cancer screening and on *Helicobacter pylori* treatment [35], and in a Caucasian random sample of the general population [36], despite showing some divergencies [37,38].

Nevertheless, our findings put the role of the T allele under a new perspective since the latest meta-analyses on these topics reported inconsistent results. In a meta-analysis carried out on 29 studies and more than 2000 individuals, Munafò and colleagues found no statistically significant difference in the A1 (T) allele frequency between former or current smokers and individuals who had never smoked. They concluded that there was no evidence of an association between this polymorphism and nicotine dependence [39].

A second meta-analysis [40] carried out on 18 studies confirmed the presence of a strong heterogeneity among the results of the studies but identified an effect played by ethnicity. Considering only the studies carried out on Caucasians, as in our study, the TT homozygosity was higher among subjects with a history of cigarette smoking. This finding was in contrast with those of other studies carried out on Asian populations, in which a greater prevalence of CC homozygosity was reported [40].

In any case, the results of our study are in contrast with the aforementioned meta-analyses, given that the presence of the T allele can protect from nicotine dependence in patients with mood disorders and schizophrenia spectrum disorders. This result could be related to the small study sample and gender composition, which could influence the effect of the DRD2 rs1800497 genotype on smoking habits [39]. Another possible explanation is that our study was carried out on patients with various psychiatric diagnoses: the surge of cigarette smoking could have a different pathophysiological basis in these patients. Furthermore, patients with mental disorders may exhibit different smoking behaviours than healthy subjects, as smoking may represent an attempt at self-medication [3].

The presence of the T allele has been associated with a lower D2 receptor availability at the striatal level [41]. Considering the fundamental role played in addictions by dopaminergic projections directed to the ventral striatum, we can hypothesize that the different expression of the receptor plays a role in nicotine consumption. Our study found low levels of nicotine dependence in patients expressing the T allele, therefore possibly showing a lower striatal expression of the receptor. Such finding could agree with the hypothesis of self-medication with nicotine by psychiatric patients. Individuals with higher receptor expression may request more nicotine for a receptor effect and a subsequent reward. Our results align with existing evidence of higher T allele frequency in healthy volunteers with a medical history, both former and current smokers [37,38].

*DRD3 rs6280*. We found that the expression of the T allele was associated with lower levels of nicotine addiction measured by administering the Fagerstrom scale, lower cigarette consumption, and fewer years of smoking in patients with treatment-resistant mood disorders and schizophrenia spectrum disorders. The existing literature on this polymorphism and its association with nicotine dependence is limited and worthy of investigation [16].

A recent review underlined how the D3 receptor might be implicated in addictions, due to both its localization in the mesolimbic dopaminergic system and its influence on reward and motivation mechanisms. This receptor plays a role in craving and searching for abuse substances [42]. This is the first study that showed the involvement of DRD3 rs6280 in nicotine dependence in patients with mental disorders. All the studies on this issue are focused on healthy people. A study on randomly recruited healthy subjects associated the presence of the C vs. T allele with a higher daily cigarette consumption and a shorter time between waking up and lighting the first cigarette [20]. A second study replicated these results in European-Americans, associating the presence of the C allele with a greater number of cigarettes smoked and a higher score on the Fagerstrom scale [23]. Another very recent study also showed greater craving for nicotine in medically and psychologically healthy subjects expressing the C allele [43].

This initial evidence on human samples is associated with further evidence from in vitro and animal studies. In vitro studies demonstrated that CC homozygosity correlated with increased dopamine affinity (up to fivefold) for the D3 receptor [44,45]. In addition, two animal studies highlighted the role of the D3 receptor in addictions, and specifically in nicotine addiction: the administration of D3 antagonists reduced the self-administration of nicotine and craving behaviour [46,47]. Our results further confirm the role of the rs6280 in the mechanisms of craving and nicotine addiction, probably in relation to changes in the dopaminergic affinity of the D3 receptor.

### Limitations

The major limitation of our study is the small sample number. However, this is the first study on genomic correlates of nicotine dependence in a relatively rare population of patients with schizophrenia spectrum disorders and severe mood disorders. Further studies on larger samples are needed to deepen the results obtained from this study.

## 5. Conclusions

DRD2 rs1800497 and DRD3 rs6280 are involved in nicotine dependence in patients with treatment-resistant mental disorders. The T allele of the rs1800497 is associated with lower nicotine consumption and dependence. The expression of the T allele of the DRD3 rs6280 polymorphism was associated with lower levels of nicotine dependence and lower cigarette consumption and years of smoking. There is a need for further studies on the involvement of these polymorphisms in the nicotine dependence of patients with treatment-resistant mental disorders. New findings could pave the road to developing new clinical applications for personalized treatment strategies.

## Figures and Tables

**Table 1:** Socio-demographics and clinical characteristics of the studied sample.

	**MDD vs. BD**					**t**	**df**	***p*** **(2-Tailed)**	**95% Confidence Interval of the Difference**	
**Diagnosis**		**N**	**Mean**	**SD**	**SE**				**Lower**	**Upper**
Age	MDD	35	50.8529	14.95677	2.56507	0.860	76	0.392	−4.14102	10.43781
	BD	43	47.7045	16.80473	2.53341					
Years of smoking	MDD	35	21.1176	21.61336	3.70666	0.987	76	0.327	−4.92909	14.61892
	BD	43	16.2727	21.39807	3.22588					
Cigarettes per day	MDD	35	9.2059	10.23594	1.75545	1.086	76	0.281	−2.03107	6.89738
	BD	43	6.7727	9.48159	1.42940					
FAG	MDD	35	2.9412	3.42841	0.58797	1.719	76	***0.090***	−0.18915	2.57151
	BD	43	1.7500	2.69474	0.40625					
	**MDD vs. SCZ**					**t**	**df**	***p*** **(2-tailed)**	**95% Confidence Interval of the Difference**	
**diagnosis**		**N**	**Mean**	**SD**	**SE**				**Lower**	**Upper**
Age	MDD	35	50.8529	14.95677	2.56507	1.693	42	0.098	−1.62566	18.53155
	SCZ	10	42.4000	8.89694	2.81346	2.220				
Years of smoking	MDD	35	21.1176	21.61336	3.70666	0.700	42	0.488	−9.63924	19.87454
	SCZ	10	16.0000	14.67424	4.64040	0.862				
Cigarettes per day	MDD	35	9.2059	10.23594	1.75545	−1.651	42	0.106	−15.10000	1.51177
	SCZ	10	16.0000	15.05545	4.76095	−1.339				
FAG	MDD	35	2.9412	3.42841	0.58797	−1.080	42	0.286	−3.89848	1.18083
	SCZ	10	4.3000	3.74314	1.18369	−1.028				
	**BD vs. SCZ**					**t**	**df**	***p*** **(2-tailed)**	**95% Confidence Interval of the Difference**	
**diagnosis**		**N**	**Mean**	**SD**	**SE**				**Lower**	**Upper**
Age	BD	43	47.7045	16.80473	2.53341	0.963	52	0.340	−5.74858	16.35768
	SCZ	10	42.4000	8.89694	2.81346	1.401				
Years of smoking	BD	43	16.2727	21.39807	3.22588	0.038	52	0.970	−14.06351	14.60897
	SCZ	10	16.0000	14.67424	4.64040	0.048				
Cigarettes per day	BD	43	6.7727	9.48159	1.42940	−2.472	52	***0.017***	−16.71891	−1.73564
	SCZ	10	16.0000	15.05545	4.76095	−1.856				
FAG	BD	43	1.7500	2.69474	0.40625	−2.507	52	***0.015***	−4.59103	−0.50897
	SCZ	10	4.3000	3.74314	1.18369	−2.038				

Legend. BD: Bipolar Disorder; FAG: Fagerstrom Test for Nicotine Dependence; MDD: Major Depressive Disorder; N: number; SD: Standard deviation; SE: Standard error. Bold italic indicates significant results for *p* < 0.05.

**Table 2:** Between-genotype comparison of nicotine dependence-related variables (significant results).

**DRD2 rs1800497**						**t**	**df**	***p***	**95% Confidence Interval of the Difference**	
		**N**	**Mean**	**SD**	**SE**				**Lower**	**Upper**
Years of nicotine dependence	T allele not expressed (C/C)	64	20.9375	20.63890	2.57986	1.990	85	***0.050***	0.00804	19.78000
	T allele expressed	23	11.0435	19.90769	4.15104					
Daily number of cigarettes	T allele not expressed (C/C)	64	10.5000	11.15120	1.39390	2.431	85	***0.017***	1.12849	11.26281
	T allele expressed	23	4.3043	8.27611	1.72569					
FAG score	T allele not expressed (C/C)	64	2.9531	3.29709	0.41214	2.100	85	***0.039***	0.08569	3.12490
	T allele expressed	23	1.3478	2.65619	0.55385					
**DRD3 rs6280**						**t**	**df**	***p***	**95% Confidence Interval of the Difference**	
		**N**	**Mean**	**SD**	**SE**				**Lower**	**Upper**
Years of nicotine dependence	T allele not expressed (C/C)	10	29.1000	21.75342	6.87903	1.763	85	0.082	−1.55672	25.91257
	T allele expressed	77	16.9221	20.40367	2.32521					
Daily number of cigarettes	T allele not expressed (C/C)	10	15.5000	13.42676	4.24591	2.111	85	***0.038***	0.43731	14.56269
	T allele expressed	77	8.0000	10.17608	1.15967					
FAG score	T allele not expressed (C/C)	10	4.7000	3.97352	1.25654	2.335	85	***0.022***	0.36430	4.54219
	T allele expressed	77	2.2468	3.00944	0.34296					

Legend. FAG: Fagerstrom Test for Nicotine Dependence; N: number; SD: Standard deviation; SE: Standard error. Bold italic indicates significant results for *p* < 0.05.

## Data Availability

Data supporting reported results can be requested from the corresponding author.

## Supplementary Materials

The following are available online at https://www.mdpi.com/article/10.3390/jpm12040565/s1, Table S1: Between-genotypes comparison of nicotine dependence related variables (all results).
