# Supplementary material for: Dopamine DRD2 and DRD3 Polymorphisms Involvement in Nicotine Dependence in Patients with Treatment-Resistant Mental Disorders
Source: J Pers Med. 2022 Apr 2;12(4):565. doi: 10.3390/jpm12040565 (PMC9033085; doi:10.3390/jpm12040565)
Supplement: Supplementary file 1 [file jpm-12-00565-s001.zip › jpm-1581685-supplementary.pdf]

Table S1. Between-genotypes comparison of nicotine dependence related variables (all results).

SLC6A4									95% Confidence Interval of the Difference	
		N	Mean	SD	SE	t	df	p	Lower	Upper
Years of nicotine dependence	S allele not expressed (L/L)	29	18.4828	20.38315	3.78506	0.071	84	0.943	-9.19993	9.88474
	S allele expressed	57	18.1404	21.35703	2.82881					
Daily number of cigarettes	S allele not expressed (L/L)	29	8.4483	9.15574	1.70018	-0.174	84	0.863	-5.34354	4.48570
	S allele expressed	57	8.8772	11.58366	1.53429					
FAG score	S allele not expressed (L/L)	29	2.5517	3.11203	0.57789	0.155	84	0.877	-1.34093	1.56718
	S allele expressed	57	2.4386	3.25147	0.43067					

SLC6A4									95% Confidence Interval of the Difference	
		N	Mean	SD	SE	t	df	p	Lower	Upper
Years of nicotine dependence	L allele not expressed (S/S)	15	19.0667	20.74838	5.35721	0.164	84	0.870	-10.90450	12.86882
	L allele expressed	71	18.0845	21.09147	2.50310					
Daily number of cigarettes	L allele not expressed (S/S)	15	10.6000	12.67055	3.27152	0.737	84	0.463	-3.84225	8.36619
	L allele expressed	71	8.3380	10.38810	1.23284					
FAG score	L allele not expressed (S/S)	15	2.9333	3.34806	0.86447	0.608	84	0.545	-1.25474	2.36085
	L allele expressed	71	2.3803	3.16844	0.37603					

DRD2 rs1800497									95% Confidence Interval of the Difference	
		N	Mean	SD	SE	t	df	p	Lower	Upper
Years of nicotine dependence	T allele not expressed (C/C)	64	20.9375	20.63890	2.57986	1.990	85	***0.05***	0.00804	19.78000
	T allele expressed	23	11.0435	19.90769	4.15104					
Daily number of cigarettes	T allele not expressed (C/C)	64	10.5000	11.15120	1.39390	2.431	85	***0.017***	1.12849	11.26281
	T allele expressed	23	4.3043	8.27611	1.72569					
FAG score	T allele not expressed (C/C)	64	2.9531	3.29709	0.41214	2.100	85	***0.039***	0.08569	3.12490
	T allele expressed	23	1.3478	2.65619	0.55385					

OPRM1 rs1799971									95% Confidence Interval of the Difference	
		N	Mean	SD	SE	t	df	p	Lower	Upper
Years of nicotine dependence	G allele not expressed (A/A)	57	20.0351	21.34576	2.82732	1.060	85	0.292	-4.35365	14.29050
	G allele expressed	30	15.0667	19.66080	3.58955					
Daily number of cigarettes	G allele not expressed (A/A)	57	9.2807	10.40219	1.37780	0.497	85	0.620	-3.64094	6.06901
	G allele expressed	30	8.0667	11.59945	2.11776					
FAG score	G allele not expressed (A/A)	57	2.4561	3.02413	0.40056	-0.290	85	0.773	-1.65583	1.23478
	G allele expressed	30	2.6667	3.57514	0.65273					

COMT rs4633									95% Confidence Interval of the Difference	
		N	Mean	SD	SE	t	df	p	Lower	Upper
Years of nicotine dependence	C allele not expressed (T/T)	15	9.6000	15.51865	4.00690	-1.521	71	0.133	-21.08805	2.83977
	C allele expressed	58	18.7241	21.80117	2.86263					
Daily number of cigarettes	C allele not expressed (T/T)	15	9.4000	14.33178	3.70045	0.308	71	0.759	-5.48841	7.49531
	C allele expressed	58	8.3966	10.33974	1.35767					
FAG score	C allele not expressed (T/T)	15	2.5333	3.79599	0.98012	0.239	71	0.812	-1.63682	2.08279
	C allele expressed	58	2.3103	3.06197	0.40206					

COMT rs4633									95% Confidence Interval of the Difference	
		N	Mean	SD	SE	t	df	p	Lower	Upper

Years of nicotine dependence	T allele not expressed (C/C)	27	16.7778	20.68134	3.98013	-0.022	71	0.982	-10.28842	10.06137
	T allele expressed	46	16.8913	21.25739	3.13423					
Daily number of cigarettes	T allele not expressed (C/C)	27	8.0370	11.11299	2.13870	-0.329	71	0.743	-6.33058	4.53509
	T allele expressed	46	8.9348	11.31057	1.66765					
FAG score	T allele not expressed (C/C)	27	2.3333	3.16228	0.60858	-0.046	71	0.963	-1.59340	1.52093
	T allele expressed	46	2.3696	3.25480	0.47989					

DRD3 rs6280									95% Confidence Interval of the Difference	
		N	Mean	SD	SE	t	df	p	Lower	Upper
Years of nicotine dependence	C allele not expressed (T/T)	35	16.8571	21.28399	3.59765	-0.537	85	0.593	-11.53065	6.62955
	C allele expressed	52	19.3077	20.61937	2.85939					
Daily number of cigarettes	C allele not expressed (T/T)	35	8.3143	9.58790	1.62065	-0.387	85	0.700	-5.62514	3.79218
	C allele expressed	52	9.2308	11.58704	1.60683					
FAG score	C allele not expressed (T/T)	35	2.6286	3.08779	0.52193	0.237	85	0.813	-1.23414	1.56821
	C allele expressed	52	2.4615	3.31048	0.45908					

DRD3 rs6280									95% Confidence Interval of the Difference	
		N	Mean	SD	SE	t	df	p	Lower	Upper
Years of nicotine dependence	T allele not expressed (C/C)	10	29.1000	21.75342	6.87903	1.763	85	*0.082*	-1.55672	25.91257
	T allele expressed	77	16.9221	20.40367	2.32521					
Daily number of cigarettes	T allele not expressed (C/C)	10	15.5000	13.42676	4.24591	2.111	85	***0.038***	0.43731	14.56269
	T allele expressed	77	8.0000	10.17608	1.15967					
FAG score	T allele not expressed (C/C)	10	4.7000	3.97352	1.25654	2.335	85	***0.022***	0.36430	4.54219
	T allele expressed	77	2.2468	3.00944	0.34296					

CYP2A6 rs1801272 and rs28399433									95% Confidence Interval of the Difference	
		N	Mean	SD	SE	t	df	p	Lower	Upper
Years of nicotine dependence	Normal metabolizers	71	18.2113	21.12271	2.50680	.386	83	.701	-9.77838	14.48663
	Slow metabolizers	14	15.8571	19.38222	5.18012					
Daily number of cigarettes	Normal metabolizers	71	9.0986	10.69734	1.26954	.276	83	.783	-5.48408	7.25269
	Slow metabolizers	14	8.2143	12.21713	3.26517					
FAG score	Normal metabolizers	71	2.5634	3.14748	.37354	.294	83	.769	-1.59923	2.15457
	Slow metabolizers	14	2.2857	3.62531	.96890					

Legend. BD: Bipolar Disorder; FAG: Fagerstrom Test for Nicotine Dependence; MDD: Major Depressive Disorder; N: number; SD: Standard deviation; SE: Standard error. Bold italic indicates significant results for $p < 0.05$.
